# A Preliminary Analysis of Ki-67 Expression in Breast Cancer in the Caribbean

**DOI:** 10.7759/cureus.38351

**Published:** 2023-04-30

**Authors:** Joshua A Jogie, Akshay Maharaj, Tarini Mahase, Sinead Bhagwandeen, Levi Ramcharan, Riyad Mohammed, Jimmy Ramdass, Vinash Deyalsingh

**Affiliations:** 1 Faculty of Medical Sciences, University of the West Indies, St. Augustine, TTO; 2 Internal Medicine, Port of Spain General Hospital, Port of Spain, TTO; 3 Ophthalmology, Port of Spain General Hospital, Port of Spain, TTO; 4 Internal Medicine, Howard University Hospital, Washington DC, USA; 5 General Surgery, Port of Spain General Hospital, Port of Spain, TTO

**Keywords:** grade, receptor, trinidad, lymph node, staging, caribbean, breast cancer, ki, ki-67

## Abstract

Objective: There has been a void of data regarding Ki-67 expression in breast cancer in the Caribbean. Ki-67 is a widely used marker to determine the grade and prognosis of breast cancer. Ki-67 has been shown to be a valuable tool in predicting the response to chemotherapy and hormonal therapy in breast cancer patients. The objective of this preliminary study aims to describe the Ki-67 (Ki) status in this population and its correlations with other parameters in breast cancer histology. This study also aims to lay the groundwork for Ki-67 analysis in this population so that future studies may better describe it.

Method: The methodology involved gathering data from histology reports for all breast cancer-related biopsies from the 1st of January 2018 to the 12th of July 2021. This data was retrospectively analyzed.

Results: Twenty-three Ki-67 cases were obtained, 19 of which had Ki expression >10%. This >10% group was mostly staged from T1c up (one T1, three T1c cases, two T2 cases, four T3 cases while nine were not T staged). Two were N2/M1 while 9 were N0 and two were M0, the rest were not staged. The mean age was 65.6 years with a range of 54 and a standard deviation of 12.5. Lymphovascular invasion was confirmed in four cases and suspected in three. Axillary lymph node dissection (ALND) yielded >10 nodes involved in two cases while <5 nodes in the remaining. The most common receptor status was hormone positive/ human epidermal growth factor receptor-2 (HER) negative (eight). Invasive Ductal Carcinoma (IDC) occurred in 10 cases while intermediate grade was in 14 cases. The Ki 6-10 % group consisted of two cases, one staged at T1aN0Mx while the other T2NxMx. Lymphovascular invasion was suspected in one. The average age was 67.5 years. ALND yielded less than five nodes in one case and 5-10 nodes in the other. Grades were high and intermediate. Histology was invasive ductal carcinoma/ductal carcinoma in situ (IDC/DCIS), and ductal carcinoma in situ ( DCIS) respectively. The Ki <6% group comprised two cases, staged at T1NxMx and T3N2M1. Lymphovascular invasion was absent in both. The mean age was 58.5 years. ALND yielded >10 nodes in one case and <5 in the other. Grades were high and intermediate. Histology was IDC/DCIS in both. There were no sentinel nodes involved in all but two cases belonging to the Ki >10% group.

Conclusion: This preliminary study was the first to describe the Ki-67 marker in the Caribbean population. The vast majority of this population has a Ki-67 level of>10%. Higher Ki-67 expression is associated with larger tumors, lymphovascular invasion, metastases, and higher tumor grades. There is a need for consistent Ki-67 reporting in histology samples before follow-up studies are conducted.

## Introduction

Breast cancer is one of the most common cancers diagnosed each year globally [1] and its incidence has been on the rise over the past few years. In 2020 there were 19 million new cases diagnosed globally with 14,712 cases diagnosed in the Caribbean alone [2]. The mortality of breast cancer, at 6.9% globally is second only to colorectal cancer at 9.4% [1]. Both the incidence and mortality were only second to prostate cancer in Trinidad and Tobago in 2020, as reported by the WHO [3]. Thus, breast cancer is a major disease with a great impact. It is paramount to study and understand it at the biological level so that it can be better managed.

Many biomarkers are used to determine prognosis and guide treatment in breast cancer. A relatively new marker is Ki-67 (Ki). Ki-67 is a nuclear antigen associated with cellular proliferation and is absent in resting cells. Thus, it can be used as a tool to determine the amount of actively proliferating cells in a tumor [4]. Although Ki-67 has been studied in developed countries, it has never been detailed in the Caribbean. This lack of information in the region should be addressed to better understand breast cancer in this population and how it differs from those in other regions.

The clinical use of Ki-67 has been limited to stage 1 and 2 breast cancers due to analytical validity and the requirement for careful attention to preanalytical issues [5]. The expression of Ki-67 is thought to be higher with certain molecular and histological subtypes of breast cancer. For instance, 60% of triple-negative tumors had Ki >15%. The patients that had Ki<15% demonstrated better survival than patients who had Ki>15% [6]. Higher Ki-67 levels have been linked with other poor prognostic factors and poorer outcomes, especially in Estrogen receptor (ER) positive tumors [5,7].

Studies have demonstrated that Ki-67 is associated with old age, high tumor grades, lymph node spread, and human epidermal growth factor receptor 2 (HER2 or HER) positivity. This study also concluded there was no statistically significant association with hormone receptor positivity, tumor size, or lymphovascular invasion [7]. Furthermore, in a study of 1951 cases of primary breast cancer at a hospital in Pakistan in 2019, triple-negative breast cancer was found to have the highest number of cases with high Ki-67 expression (>45%) while the Luminal A subtype had the lowest number of cases, Furthermore, metaplastic breast cancer was determined to possess a higher Ki-67 level compared to ductal carcinoma [8]. In another study of 107 patients with invasive breast cancer at an oncology center in Egypt, 33.8% of patients were found to have significant Ki-67 levels, again with the highest levels of expression seen amongst patients with triple-negative breast cancer [6]. Similarly, a large study of 3,658 patients from an oncology center in Bavaria showed a prevalence of 43% of clinically significant levels of Ki-67 expression, with higher levels being associated with both lower disease-free and overall survival rates [9].

The prognostic relevance of Ki-67 is apparent where higher levels were found to be associated with pathological complete responses to neoadjuvant therapy regardless of the breast cancer receptor type [10]. This was seen again in a group of 945 triple negative breast cancer patients where overall survival was improved in the high Ki-67 group. However, this study also reported that Ki-67 >30% was significantly associated with worse prognosis, especially in T1 patients [11]. The inverse association of higher Ki-67 with less hormone receptor expression was affirmed in a study in Indonesia [12].

## Materials and methods

Institutional Review Board approval was not required for the commencement of this retrospective study. This study took place at Port of Spain General Hospital, Trinidad. The histology department medical record system was accessed. The system was searched for the phrase 'breast cancer.' A list of all histology reports for preoperative breast cancer biopsies were generated. All histology reports from the 1st of January 2018 to the 12th of July 2021 were printed. This generated a sample size of 765 female patients. Each report was assigned a unique identifier. Various fields of data were extracted from the reports including: patient age, ethnicity, clinical presentation, procedures performed, staging, size of nodal metastases, histology type, invasion of margins, subtype, tumor grade, differentiation, presence of lymphovascular invasion, extra nodal extension, Allred score, Ki-67 level, receptor status, number of sentinel lymph nodes biopsied, number of axillary lymph nodes involved and if neoadjuvant therapy was done. The number of axillary lymph nodes was categorized and recorded as either less than 5 nodes, 5-10 nodes or more than 10 nodes.

These fields were assigned a numerical code for each possibility. For instance, African patients were assigned the number '1' and Indian patients were assigned number '2.' These numerical codes were entered and tabulated into a spreadsheet. The spreadsheet was then analyzed using IBM SPSS Statistics software, version 28.0 (IBM Corp., Armonk, NY). The patients who did not have their Ki-67 reported on histology were excluded from the analysis. This generated a population of 23 patients with Ki-67 expression. SPSS was used to calculate the mean age along with its standard deviation. SPSS was also used to generate frequency counts and percentages on each data field extracted from these 23 cases. All the 23 cases were then split into three groups based on their level of Ki expression being less than 6%, 6-10%, and greater than 10%. SPSS was utilized again to calculate mean age and standard deviation along with frequency counts and percentages for each group separately. These results were inferred against a background of Ki-67 research done previously.

## Results

**Figure 1:**
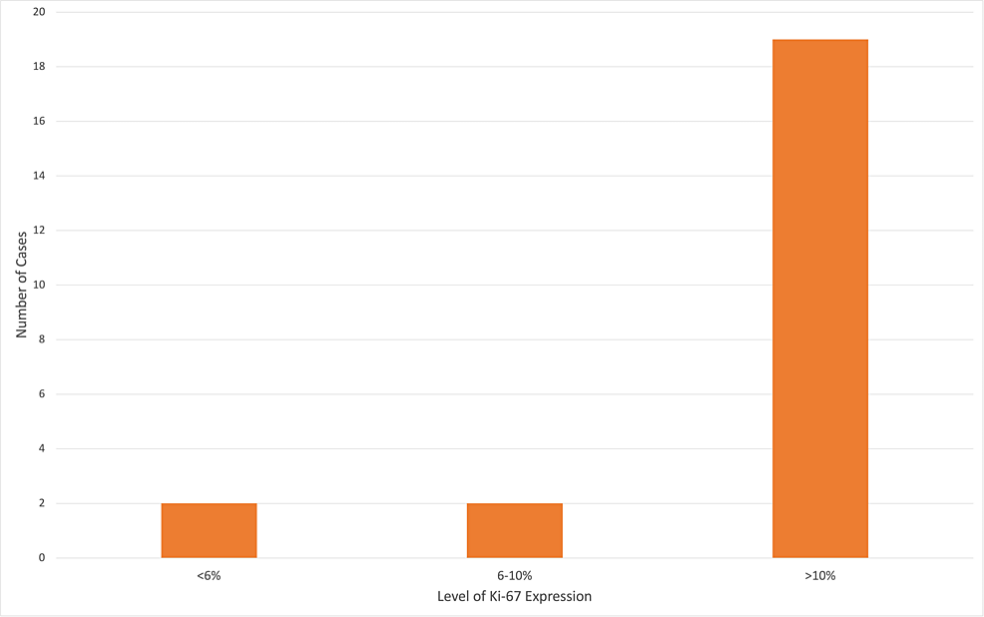
Level of Ki-67 Expression in the Sample Used

There were 23 cases of the Ki-67 (Ki) expression documented from 765 patients over three years. This corresponds to a 3% reporting rate of Ki-67 at Port of Spain General Hospital. As seen in Figure 1 below, there were 19 cases (82.6%) with Ki>10%, two cases (8.7%) with Ki <6% and two cases (8.7%) with Ki 6-10%.

The Ki >10% group was mostly staged from T1c up (three T1c cases, two T2 cases, four T3 cases while nine were not T staged). Two were N2/M1 while nine were N0 and two were M0 as shown in Table 1. The rest were not staged. The mean age was 65.6 years with a range of 54 and a standard deviation of 12.5. Lymphovascular invasion was confirmed in four cases and suspected in three. Axillary lymph node dissection (ALND) yielded >10 nodes involved in two cases and less than five nodes in the remaining. The most common receptor status was hormone positive/human epidermal growth factor receptor 2 (HER2) negative (eight cases). According to Table 1, infiltrating ductal carcinoma (IDC) occurred in 10 cases and IDC/Ductal Carcinoma In Situ occurred in five cases. Intermediate grade was observed in 14 cases. 

**Table 1:** Contrasting Various Parameters Based on the Level of Ki-67 Expression IDC = Invasive Ductal Carcinoma; T= Tumor; N= Nodal; M= Metastases

	Collective Ki Cases	Ki <6%	Ki 6-10%	Ki>10%
Distribution of Cases				
Cases	23	2	2	19
T stage				
T1	2	1	0	1
T1a	1	0	1	0
T1c	3	0	0	3
T2	3	0	1	2
T3	5	1	0	4
N stage				
N0	8	0	1	7
N2	3	1	0	2
M stage				
M0	2	0	0	2
M1	3	1	0	2
Margins IDC				
Involved	4	1	0	3
Histology				
Invasive Ductal Carcinoma only	10	0	0	10
Invasive Ductal Carcinoma & Ductal Carcinoma in Situ	8	2	1	5
Invasive Lobular Carcinoma only	1	0	0	1
Ductal Carcinoma in Situ only	1	0	1	0
Subtype				
Comedo	5	0	0	5
Mucoid	2	1	0	1
Cribriform	1	0	1	0
Grade				
intermediate	15	1	0	14
high	7	1	1	5
Lymphovascular invasion				
present	5	1	0	4
Suspicious	4	0	1	3
Number of Sentinel Lymph Nodes Biopsied				
0 nodes	21	2	2	17
1 node	2	0	0	2
Number of Nodes involved in Axillary Lymph Node Dissection				
<5 nodes	19	1	1	17
5-10 nodes	1	0	1	0
>10 nodes	3	1	0	2

The Ki 6-10 % group consisted of two cases, one staged at T1aN0Mx while the other T2NxMx. Lymphovascular invasion was suspected in one case. The average age was 67.5 years. ALND yielded less than five nodes in one case and 5-10 nodes in the other. Grades were high and intermediate respectively. Histology was mixed IDC/ductal carcinoma in situ (DCIS) and DCIS respectively.

The Ki <6% group comprised two cases staged at T1NxMx and T3N2M1. Lymphovascular invasion was present in one case. The mean age was 58.5 years. ALND yielded >10 nodes in one case and less than five nodes in the other as seen in Table 1 above. Grades were high and intermediate. The histology was found to be mixed IDC/DCIS in both cases.

For the collective Ki-67 group, the patient ages ranged from 34 to 88 years of age with the mean age being of 65 years of age. Ki-67 was expressed most frequently in the 51-60 (eight cases) and 61-70 (eight cases) age groups. This was followed by the 71-80 (three cases) and 81-90 (three cases) age groups and lastly the 31-40 (one case) age groups. The 51-60 (eight cases) group was then further broken down with Ki > 10% having the greatest number of cases (six cases) followed by Ki 6-10% (one case) and Ki<6% (one case). The 61-70 (eight cases) age group was further broken down into Ki >10% (seven cases) followed by Ki <6% (one case). No cases were reported for Ki 6-10% in the age group of 61-70.

Conversely, the age group with the least prevalence was the 31-40 (one case) age group with the one case occurring in the Ki >10% group. Both the 71-80 (three cases) and 81-90 (three cases) age groups had the second least number of cases at three cases each. They were further subdivided into 71-80 years containing (two cases) Ki>10%, (one case) Ki 6-10%, and the 81-90 years group containing all cases Ki>10%. 

Moreover, the procedure type most utilized was a mastectomy and axillary clearance (eight cases) which was broken down into (seven cases) in Ki >10% and (one case) in Ki <6%. This was then followed by mastectomy (six cases) which was broken down into (five cases) for Ki >10%, and (one) for Ki 6-10%. Both wide local excision and trucut had four cases each which were subdivided into two cases Ki >10%, one case Ki 6-10% and one case Ki <6% for wide local excision. Lastly, wide local excision and axillary clearance was the least utilized procedure type with one case which fell under Ki >10%.

Additionally, T staging analysis revealed that the majority of nine cases fell under the category of not reported, five cases under T3, three cases each under T1c and T2 respectively, two cases under T1, and finally one case for T1a. The not done category consisted of Ki >10% cases. T3 was subdivided into four cases Ki >10% and one case Ki <6%. T1c tumors were all Ki>10% and T2 three cases >10% and one case Ki<6%. T1 had one case each for Ki <6% and Ki >10% and lastly, T1a was a single case in Ki 6-10%.

N staging revealed Nx having the majority of cases (12) which were further subdivided into (one) Ki<6%, (one) Ki 6-10%, and (10) Ki >10%. This was followed by N0 (eight) which was subdivided into (one) Ki 6-10% and (seven) Ki >10%. Lastly, N2 had the least number of cases (three) which were subdivided into (one) Ki <6% and (two) Ki >10%. Additionally, the size of nodal metastases was interestingly not mentioned for 22 of the cases which were further subdivided into (one) Ki<6%, (two) Ki 6-10%, and (19) Ki >10%, and one case was that of a macro metastasis falling under the category of Ki <6%.

Also, M staging revealed that the majority of cases fell under not mentioned (16), followed by M1 (three) then M0 and Mx both with two cases each. Not mentioned was further subdivided into (14) Ki >10%, (one) Ki 6-10%, and (one) Ki <6%. M1 was further subdivided into (two) >10% and (one) <6%. M0 and Mx were further subdivided into (two) >10% for M0 while Mx was (one) >10% and (one) 6-10%.

To add to this, the histology results revealed that IDC was the most common at 10 cases followed by IDC and DCIS (eight), other (three), invasive lobular carcinoma (ILC) only (one) and DCIS only (one). IDC was further subdivided into all cases falling under Ki>10%. IDC and DCIS were subdivided into (five) Ki >10%, (two) Ki <6%, and (one) Ki 6-10%. Other consisted solely of Ki >10%. ILC consisted of (one) Ki >10% and DCIS only (one) Ki 6-10%.

In addition, the margins of invasion showed that the majority (15) were free, and both involved and not specified had four cases each. Free margins were subdivided into (12) >10%, (two cases) 6-10 %, and (one case) <6%. Involved was divided into (three) >10% and (one) < 6%. Lastly, not specified was subdivided into all four cases being >10 %. Additionally, the margin status of ductal carcinoma in situ revealed (20) not specified, (two) >2mm, and (one) no ink on the tumor. Not specified was subdivided into (one) <6%, (two) 6-10%, and (17) >10%. The >2mm cases showed (one) <6% and (one) >10%. Finally, no ink on the tumor was Ki>10%.

Also, the subtypes for collective Ki cases were divided into not mentioned (nine), other (six), comedo (five), mucoid (two), and cribriform (one). Not mentioned was subdivided into (eight) >10% and (one) 6-10%, other (five) >10%, (one) <6%, comedo all >10%, mucoid (one) >10% and (one) <6%. Lastly, cribriform has one case in 6-10%.

The breast cancer grades showed intermediate (15), high (seven), and not mentioned (one). These were further subdivided into intermediate (14) >10% and (one) <6%, high (five) >10%, (one) 6-10%, (one) <6% and not mentioned (one) 6-10%. Additionally, the cell differentiation interestingly showed (22) not mentioned and (one) moderate. This was then subdivided into not mentioned (18) >10%, (two) 6-10%, (two) <6%, and moderate >10%.

Lymphovascular invasion revealed absent (11) which was the most prominent followed by present (five), suspicious (four), and not mentioned (three). These were further subdivided into absent (nine) >10 %, (one) 6-10% and (one) <6%, present (four) >10%, (one) < 6%, suspicious (three) >10%, (one) 6-10% and not mentioned with all cases >10%. Moreover, it was revealed that all the cases showed extranodal extension (23). This was further subdivided into (19) >10%, (two) 6-10%, and (two) <6%.

**Figure 2:**
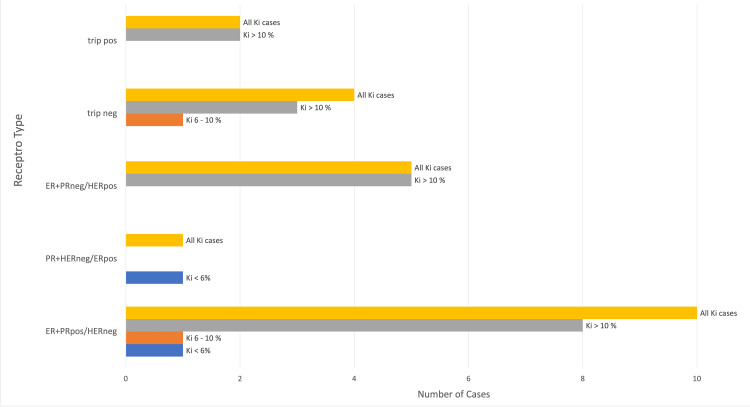
Receptor Status for Infiltrating Ductal Carcinoma for Ki <6%, 6-10%, Ki >10 % and all Ki cases trip pos = triple positive receptors; trip neg= triple negative receptors; ER = Estrogen Receptor; PR = Progesterone Receptor; HER = Human Epidermal Growth Factor Receptor 2; pos = positive; neg = negative

Also, the total number of Ki cases analyzed for receptor status amounted to 22 as there was one case in which the receptor status was unreported. The receptor status for infiltrating ductal carcinoma revealed that the most commonly occurring status was estrogen receptor (ER)/progesterone receptor (PR) positive and HER2 negative (10) followed by ER/PR negative and HER2 positive (five), triple-negative (four), triple-positive (two) and PR/HER2 negative and ER positive (one). These were then further subdivided into ER/PR positive and HER2 negative (eight) >10%, (one) 6-10 %, (one) <6%, ER/PR negative and HER2 positive all >10%, triple negative (three) >10%, (one) 6-10%, triple positive (two) <10% and finally PR/HER2 negative and ER positive <6%. These findings can be seen as illustrated in Figure 2 below.

Additionally, the receptor status for ductal carcinoma in situ showed that the greatest number of cases occurred in not mentioned (13), ER/PR positive (four), ER/PR negative (four), and ER-negative/ PR positive (one). These were then further subdivided into not mentioned all >10%, ER/PR positive (two) >10%, (one) 6-10%, (one) <6%, ER/PR negative (three) >10%, (one) 6-10% and ER-negative/PR positive <6%. None of the cases were HER2 positive and therefore fluorescence in situ hybridization (FISH) was not done for any of the cases.

The Allred scores for the cases revealed that the majority (22) cases were not specified and (one) was 0-2. These were further subdivided into not specified cases (18) >10%, (two) 6-10%, (two) <6%, and the 0-2 case was Ki >10%. Also, the H score was not specified for all 23 cases. In addition, the Nottingham scores for three cases showed scores of five, six, and seven respectively. Scores five and six were Ki <6% while score seven was Ki >10%. No extensive intraductal component was noted in any of the cases.

To add to this, neoadjuvant therapy was not recorded for the majority of cases (20) and was done for three cases. Of the cases where neoadjuvant therapy was not recorded (17) were ki <10%, (two) <6%, and (one) 6-10%. The three cases where neoadjuvant therapy was done were subdivided into (one) 6-10 % and (two) >10%

The numbers of sentinel lymph nodes biopsied were one each for two cases and the majority were not biopsied (21). These were further subdivided with the two cases biopsied both being Ki >10% and of the not biopsied cases (17) were >10%, (two) 6-10%, and (two) <6%. To add to this, the number of axillary nodes biopsied showed the majority (19) <5 nodes, (three) >10 nodes, and (one) 5-10 nodes. These were further subdivided into <5 nodes (17) >10%, (one) 6-10%, (one) <6%, 5-10 nodes broken down into (one) 6-10%, and lastly >10 nodes (two) >10% and (one) <6%.

## Discussion

The use of Ki-67 (Ki) nuclear antigen as a marker for breast cancer prognosis is a relatively new study worldwide with the controversy surrounding whether it can be successfully utilized in making decisions for the treatment of Breast Cancer. Ki-67 has been studied in developed countries; however, it has never been studied in the Caribbean thus far.

From the generated results, it can be inferred that higher Ki-67 levels are linked with breast cancer patients of an older age (Eta= 0.322). This is supported by the Ki >10% and 6-10% groups having a higher mean age (65.6 and 67.5 years respectively) compared to Ki <6% (58.5 years). This association is supported by a previous study [7].

Higher Ki expression was associated with larger tumors as seen where Ki >10% involved mostly T3 (four cases) and T2 (two cases) tumors whereas Ki <6% involved one case belonging to T3 and one case at T1 (Pearson Chi-square= 45.9, p value = 0.00. This is consistent with the molecular science regarding Ki-67 and its impact on cellular proliferation [4]. It also contrasts a study that failed to show Ki-67 levels are related to tumor size [7].

The Ki>10% group had more advanced N staging compared to the Ki <6% and Ki 6-10% groups. Thus, it can be stated that a higher Ki-67 is associated with more lymph node involvement. This is bolstered by the Ki >10% group having the highest number of axillary lymph nodes involved in the axillary lymph node dissection as seen in Table 1. Similarly, the presence of metastases and lymphovascular invasion was noted to be higher in the Ki >10% group compared to the other Ki groups. This is consistent with known literature where a study noted that significant Ki-67 upregulation was found in metastatic deposits compared to the primary tumor [13].

All the cases expressing Ki-67 demonstrated extranodal extension. Hence Ki expression may result in a more aggressive form of breast cancer with a propensity to spread. The presence of a high Ki-67 index in a breast biopsy can therefore suggest the degree of spread and prognosis of the patient. This is consistent with the pathophysiology of proliferating tumors and supports the other metastatic-related findings of this study.

There was a strong link between Ki levels and tumor grades with the majority of high-grade tumors belonging to the Ki> 10% group. Moreover, none of the tumors that stained positive for Ki-67 were low-grade. This is consistent with known research. One study determined that tumors being of grade two or three was predictive for a high Ki index >20% [14]. This reaffirms the suggestion of Ki-67 being a poor prognostic marker and being associated with more aggressive tumors.

Overall triple-positive, triple-negative, and human epidermal growth factor-2 (HER or HER2) positive tumors were strongly associated with higher Ki levels as seen in Figure 2. These groups had no cases with Ki <6% and comprise mainly Ki >10%. In contrast, hormone positive /HER negative tumors had weaker Ki expression since this receptor type only comprised Ki <6% cases. The association of Ki-67 and HER2 has been well established, with studies that found lower Ki levels in HER2 negative tumors going on to call it an unexpected result [15]. Overall, the trend is that higher Ki levels are associated with negative receptors on tumors.

This preliminary study aims to lay the groundwork and structure for follow-up analysis to delineate further the associations Ki-67 has in breast cancer in the Caribbean. This study was limited by the low reporting rate of Ki-67 on histology reports. It is strongly recommended for Pathology Departments to ensure consistent Ki-67 reporting in future biopsies. This will create a greater sample size for future studies to utilize and draw stronger inferences. Otherwise, the sample size will remain limited and hinder future research on this topic. There also needs to be a standard for reporting histology samples whereby other parameters such as TNM staging, grade, subtype, and receptor status are mentioned for all cases. Additionally, future studies should consider different cut off values for Ki-67 than the ones chosen in this study. For instance, Ki >20% can be studied in future research to further stratify the impact of higher Ki expression. This would be particularly relevant in this population as most patients presented with Ki>10%. Another recommendation for future studies is to collect data on clinical outcomes of these breast cancer patients.

## Conclusions

This preliminary study was the first to describe the Ki-67 marker in the Caribbean population. The vast majority (82.6%) of this population has a Ki-67 level greater than 10%. Higher Ki-67 expression is associated with larger tumors, lymphovascular invasion, metastases, higher tumor grades, and older age. Higher Ki-67 expression was also associated with tumor receptor-negative status. This study was limited by a 3% reporting rate of Ki-67. Hence there needs to be consistent Ki-67 reporting in histology samples before follow-up studies are conducted.

## References

[1] (2021). All cancers cancer incidence and mortality statistics worldwide and by region incidence mortality. https://gco.iarc.fr/today/data/factsheets/cancers/39-All-cancers-fact-sheet.pdf.

[2] (2021). Cancer incidence and mortality statistics worldwide and by region incidence mortality. https://gco.iarc.fr/today/data/factsheets/cancers/20-Breast-fact-sheet.pdf.

[3] Cours Albert Thomas (2021). Ranking (Breast), estimated number of deaths in 2020, all ages, Caribbean Hub. Ranking (Breast), estimated number of deaths in 2020, all ages, Caribbean Hub.

[4] Gerdes J, Schwab U, Lemke H, Stein H (1983). Production of a mouse monoclonal antibody reactive with a human nuclear antigen associated with cell proliferation. Int J Cancer.

[5] Nielsen TO, Leung SC, Rimm DL (2021). Assessment of Ki67 in breast cancer: updated recommendations from the International Ki67 in Breast Cancer Working Group. J Natl Cancer Inst.

[6] Soliman NA, Yussif SM (2016). Ki-67 as a prognostic marker according to breast cancer molecular subtype. Cancer Biol Med.

[7] Elkablawy MA, Albasri AM, Mohammed RA, Hussainy AS, Nouh MM, Alhujaily AS (2016). Ki67 expression in breast cancer. Correlation with prognostic markers and clinicopathological parameters in Saudi patients. Saudi Med J.

[8] Hashmi AA, Hashmi KA, Irfan M (2019). Ki67 index in intrinsic breast cancer subtypes and its association with prognostic parameters. BMC Res Notes.

[9] Inwald EC, Klinkhammer-Schalke M, Hofstädter F, Zeman F, Koller M, Gerstenhauer M, Ortmann O (2013). Ki-67 is a prognostic parameter in breast cancer patients: results of a large population-based cohort of a cancer registry. Breast Cancer Res Treat.

[10] Chen X, He C, Han D (2017). The predictive value of Ki-67 before neoadjuvant chemotherapy for breast cancer: a systematic review and meta-analysis. Future Oncol.

[11] Zhu X, Chen L, Huang B (2020). The prognostic and predictive potential of Ki-67 in triple-negative breast cancer. Sci Rep.

[12] Husni Cangara M, Miskad UA, Masadah R, Nelwan BJ, Wahid S (2021). Gata-3 and KI-67 expression in correlation with molecular subtypes of breast cancer. Breast Dis.

[13] Park D, Kåresen R, Noren T, Sauer T (2007). Ki-67 expression in primary breast carcinomas and their axillary lymph node metastases: clinical implications. Virchows Arch.

[14] Brown J, Scardo S, Method M (2022). A real-world retrospective study of the use of Ki-67 testing and treatment patterns in patients with HR+, HER2- early breast cancer in the United States. BMC Cancer.

[15] Kurbel S, Dmitrović B, Marjanović K, Vrbanec D, Juretić A (2017). Distribution of Ki-67 values within HER2 &amp; ER/PgR expression variants of ductal breast cancers as a potential link between IHC features and breast cancer biology. BMC Cancer.

