# Correction: A Preliminary Analysis of Ki-67 Expression in Breast Cancer in the Caribbean

**DOI:** 10.7759/cureus.c115

**Published:** 2023-05-09

**Authors:** Joshua A Jogie, Akshay Maharaj, Tarini Mahase, Sinead Bhagwandeen, Levi Ramcharan, Riyad Mohammed, Jimmy Ramdass, Vinash Deyalsingh

**Affiliations:** 1 Faculty of Medical Sciences, University of the West Indies, St. Augustine, TTO; 2 Internal Medicine, Port of Spain General Hospital, Port of Spain, TTO; 3 Ophthalmology, Port of Spain General Hospital, Port of Spain, TTO; 4 Internal Medicine, Howard University Hospital, Washington DC, USA; 5 General Surgery, Port of Spain General Hospital, Port of Spain, TTO

This article has been corrected to add Joshua A. Jogie as first author. Dr. Jogie was omitted from the author list. The authors greatly regret this error.

